# Dexamethasone Induces Changes in Osteogenic Differentiation of Human Mesenchymal Stromal Cells via *SOX9* and *PPARG*, but Not *RUNX2*

**DOI:** 10.3390/ijms22094785

**Published:** 2021-04-30

**Authors:** Elena Della Bella, Antoine Buetti-Dinh, Ginevra Licandro, Paras Ahmad, Valentina Basoli, Mauro Alini, Martin J. Stoddart

**Affiliations:** 1 AO Research Institute Davos, 7270 Davos Platz, Switzerland; elena.dellabella@aofoundation.org (E.D.B.); docparas2017@gmail.com (P.A.); valentina.basoli@aofoundation.org (V.B.); mauro.alini@aofoundation.org (M.A.); 2 Laboratory of applied microbiology (LMA), Department of Environment, Constructions and Design (DACD), University of Applied Sciences of Southern Switzerland (SUPSI), 6500 Bellinzona, Switzerland; antoine.buetti@supsi.ch; 3 Swiss Institute of Bioinformatics, Quartier Sorge—Batiment Genopode, 1015 Lausanne, Switzerland; 4 Dalle Molle Institute for Artificial Intelligence (IDSIA), University of Italian Switzerland (USI), 6928 Manno, Switzerland; ginevra.licandro@idsia.ch; 5 University of Applied Science and Art of Southern Switzerland (SUPSI), 6928 Manno, Switzerland; 6 Department of Orthopedics and Trauma Surgery, Medical Center—Albert-Ludwigs-University of Freiburg, Faculty of Medicine, Albert-Ludwigs-University of Freiburg, 79106 Freiburg, Germany

**Keywords:** Osteogenesis, glucocorticoids, transcription factors, MSC, gene expression, approximate Bayesian computation (ABC)

## Abstract

Despite the huge body of research on osteogenic differentiation and bone tissue engineering, the translation potential of in vitro results still does not match the effort employed. One reason might be that the protocols used for in vitro research have inherent pitfalls. The synthetic glucocorticoid dexamethasone is commonly used in protocols for trilineage differentiation of human bone marrow mesenchymal stromal cells (hBMSCs). However, in the case of osteogenic commitment, dexamethasone has the main pitfall of inhibiting terminal osteoblast differentiation, and its pro-adipogenic effect is well known. In this work, we aimed to clarify the role of dexamethasone in the osteogenesis of hBMSCs, with a particular focus on off-target differentiation. The results showed that dexamethasone does induce osteogenic differentiation by inhibiting *SOX9* expression, but not directly through *RUNX2* upregulation as it is commonly thought. Rather, *PPARG* is concomitantly and strongly upregulated, leading to the formation of adipocyte-like cells within osteogenic cultures. Limiting the exposure to dexamethasone to the first week of differentiation did not affect the mineralization potential. Gene expression levels of *RUNX2*, *SOX9*, and *PPARG* were simulated using approximate Bayesian computation based on a simplified theoretical model, which was able to reproduce the observed experimental trends but with a different range of responses, indicating that other factors should be integrated to fully understand how dexamethasone influences cell fate. In summary, this work provides evidence that current in vitro differentiation protocols based on dexamethasone do not represent a good model, and further research is warranted in this field.

## 1. Introduction

Bone is the second most transplanted tissue after blood [1]. Autologous grafting is the gold-standard treatment for bone replacement when bone healing capability is impaired. Nonetheless, its application can be limited by several factors and complications [2]. The use of bone substitutes therefore has a great clinical need, but despite the research and effort there is still a poor translational potential of in vitro results to in vivo studies in the field of biomaterial testing for bone regeneration [3]. This urges the need to go back to the bench and reconsider the way in which osteogenic differentiation and bone are investigated in vitro.

Glucocorticoids are powerful immunomodulatory and anti-inflammatory drugs, widely prescribed for the treatment of idiopathic conditions with a strong inflammatory component such as chronic obstructive pulmonary disease, rheumatoid arthritis, inflammatory bowel disease, and autoimmune disorders [4]. The long-term use of such drugs, however, comes at the expense of serious side effects, such as osteoporosis [5,6] and osteonecrosis [7,8]. Glucocorticoids exert profound effects on bone and are crucial for human osteoblast differentiation, along with the formation of the extracellular matrix [9]. Moreover, they play a vital role in controlling skeletal development and maintaining healthy bone. Despite clear induction of osteogenesis by glucocorticoids in vitro, they are still considered to be negative regulators of osteogenesis [10]. Glucocorticoids become most harmful to bone at supra-physiological levels and lead to skeletal fragility [11]. Although all bone cells are affected by high levels of glucocorticoids, rodent models reliably demonstrate that the essential targets of glucocorticoids in the skeleton are osteocytes and osteoblasts [11]. In particular, the viability of the former is predominantly associated with bone strength and is readily compromised by glucocorticoids at supraphysiological levels [12]. This mechanism of action has eventually led to the investigation of novel drug intervention approaches with osteoblast-targeted agents. Emerging osteoanabolic strategies are specifically suited to offset the detrimental effects of therapeutic glucocorticoids on the structural and molecular levels [13].

The synthetic glucocorticoid dexamethasone (dex) is commonly used in vitro in trilineage differentiation protocols for mesenchymal stromal cells (MSCs). In bone research, dex has been used for osteogenic differentiation of bone-marrow-derived MSCs (BMSCs) for almost 30 years [14], and differentiation cocktails containing at least 10 nM dex, together with ascorbic acid and a source of phosphate groups, are the standard in the field [15,16,17]. Dex has been reported to increase the expression of *RUNX2* [15,18], which is considered the master regulator of osteogenic differentiation [19]. However, the precise mechanism by which dex induces osteogenic differentiation might be cell-type-dependent, as in cells from dental follicle or dental pulp it seems to rely on *ZBTB16* but not on *RUNX2* [20,21]. It has also been reported that an initial burst of high-dose dex (100 nM for the first week of differentiation) could improve osteogenesis and reduce variability between donors [15,22]. Even though the use of dex seems to be necessary to address BMSCs to an osteogenic lineage and to achieve a good level of mineralization and differentiation in vitro [23], it has the main pitfall of repressing osteocalcin expression and inhibiting terminal differentiation [24,25]. One main mechanism of glucocorticoid-mediated osteocalcin transcriptional repression is achieved through an Egr/Krox site [25]. More recently, it has been observed that dex regulates histone deacetylase 6 (*HDAC6*) expression, and that a glucocorticoid receptor (GR)-HDAC6 repressor complex occupies both proximal and distal regions of the osteocalcin promoter, leading to the inhibition of osteocalcin transcription [24].

The glucocorticoid receptor pathway is complex and wide, and we previously demonstrated an effect of dex on human BMSC non-coding RNA expression which was independent of the differentiation status [26]. Among other side effects, dex is known to possess a pro-adipogenic effect, indeed it was demonstrated to promote adipose tissue at the expense of bone formation in bone marrow [10]. In murine cells, it was observed that dex may lead to heterogeneous osteogenic/adipogenic differentiation [27]. Notably, it was also suggested that osteoblasts with a high *PPARG* expression may switch fate and become adipocytes [28].

For these reasons, the aim of this study was to clarify the role of dex during the osteogenic differentiation of human BMSCs (hBMSCs), with a particular focus on simultaneous adipogenic commitment. The effect of dex on the three main lineage-specific transcription factors for hBMSCs, namely *RUNX2*, *SOX9*, and *PPARG* [29], was studied, with a main focus on early differentiation (day 7), which is when key lineage decisions are made [30,31].

## 2. Results

### 2.1. Dose-Dependent Effect of Dex on Differentiation

**Figure 1:**
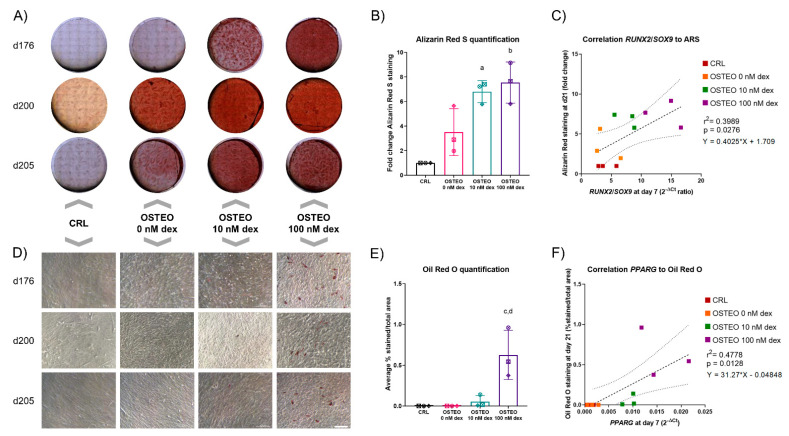
Differentiation potential of hBMSCs from three donors, using different concentrations of dex (0, 10, 100 nM). (**A**) Alizarin Red S (ARS) staining after 21 days of osteogenic differentiation, showing an increase in calcium incorporation. Images represent mosaics of pictures, transmitted light, and brightfield images. The diameter of Thermanox coverslips, which were fully pictured, was 22 mm. (**B**) ARS staining quantification was performed via elution of the stain from the cultures and the subsequent spectrophotometric determination of optical density. For each donor (represented by a different symbol), the results are expressed as a fold-change in intensity of staining compared to the undifferentiated controls. Summary of results from a two-way Analysis of Variance (ANOVA) with Tukey post-hoc test. a: *p* < 0.05; Osteo 10 nM dex vs. CRL. b: *p* < 0.01; Osteo 100 nM dex vs. CRL. (**C**) *RUNX2*/*SOX9* ratio correlation to ARS. Pearson’s correlation was calculated to correlate the *RUNX2*/*SOX9* ratio (2^-ΔCt^ ratio) at day 7 with the amount of Alizarin Red staining at day 21, expressed as a fold-change in comparison to the undifferentiated control. The correlation was found to be significant, with a *p*-value of 0.0276, pointing towards a higher calcium deposition with higher *RUNX2*/*SOX9* levels. The R^2^ value obtained (0.39898) suggests other factors were involved in the process, which influenced the linear correlation. The dashed line represents the line of best fit, of which the equation is reported. The dotted lines represent the 95% confidence interval. (**D**) Oil Red O staining after 21 days of osteogenic differentiation, showing a substantial increase in adipogenic differentiation with 100 nM dex. Scale bar = 200 µm (bottom-right image). (**E**) Oil Red O staining quantification performed via image analysis with ImageJ. The results are expressed as the average % stained area of the total area of the field. Each determination is the average of 10 different fields for each condition. Summary of results from a two-way ANOVA with the Tukey post-hoc test. c: *p* < 0.01; Osteo 100 nM dex vs. CRL and vs. Osteo 0 nM dex. d: *p* < 0.05; Osteo 100 nM dex vs. Osteo 10 nM dex. (**F**) Correlation of *PPARG* expression at day 7 with Oil Red O staining at day 21. The results show a positive correlation between the two factors. The dashed line represents the line of best fit, of which the equation is reported. The dotted lines represent the 95% confidence interval.

We first analyzed the effect of dex on osteogenic differentiation, with a particular focus on mineralization and on the formation of lipid-droplet-containing cells. After 21 days in an osteogenic-supporting environment (i.e., containing ascorbic acid and glycerol 2-phosphate), the presence of dex showed two separate effects. First, it increased calcium deposition, as expected (Figure 1A,B). In one donor, the presence of an osteogenic environment alone was sufficient alone to boost mineralization, which was not improved further by dex. The *RUNX2*/*SOX9* ratio at day 7 was shown to positively correlate with Alizarin Red staining at day 21, with a suggested dex dose-dependency (Figure 1C). In parallel, osteogenic cultures from the same donors were stained with Oil Red O to determine the presence of lipid droplets. The results showed that the increase in dex concentration concurrently led to an increase in the presence of pre-adipocyte-like cells, in particular for the 100 nM dose (Figure 1D,E). Analogously to the correlation between *RUNX2*/*SOX9* and mineralization, *PPARG* expression at day 7 positively correlated with the % area covered by Oil Red-stained cells at day 21 (Figure 1F).

**Figure 2:**
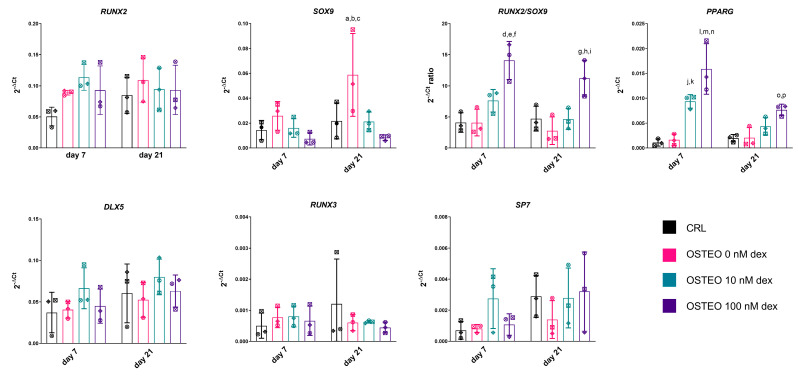
Expression of genes encoding for transcription factors with a known influence on osteogenic or adipogenic differentiation. Day 7 and day 21 data in undifferentiated controls and osteogenic commitment with 0, 10, and 100 nM dex are reported. Summary of results from a two-way ANOVA with the Tukey post-hoc test. *SOX9*. a: *p* < 0.05; Osteo 0 nM dex vs. CRL at day 21. b: *p* < 0.05; Osteo 0 nM dex vs. Osteo 10 nM dex at day 21. c: *p* < 0.01; Osteo 0 nM dex vs. Osteo 100 nM dex at day 21. *RUNX2/SOX9*. d: *p* < 0.001; Osteo 100 nM dex vs. CRL at day 7. e: *p* < 0.001; Osteo 100 nM dex vs. Osteo 0 nM dex at day 7. f: *p* < 0.05; Osteo 100 nM dex vs. Osteo 10 nM dex at day 7. g: *p* < 0.05; Osteo 100 nM dex vs. CRL at day 21. h: *p* < 0.01; Osteo 100 nM dex vs. Osteo 0 nM dex at day 21. i: *p* < 0.05; Osteo 100 nM dex vs. Osteo 10 nM dex at day 21. *PPARG*. j: *p* < 0.01; Osteo 10 nM dex vs. CRL at day 7. k: *p* < 0.01; Osteo 10 nM dex vs. Osteo 0 nM dex at day 7. l: *p* < 0.0001; Osteo 100 nM dex vs. CRL at day 7. m: *p* < 0.0001; Osteo 100 nM dex vs. Osteo 0 nM dex at day 7. n: *p* < 0.05; Osteo 100 nM dex vs. Osteo 10 nM dex at day 7. o: *p* < 0.05; Osteo 100 nM dex vs. CRL dex at day 21. p: *p* < 0.05; Osteo 100 nM dex vs. Osteo 0 nM dex at day 21. *RUNX2*, *DLX5*, *RUNX3*, *SP7*: n.s. Each symbol corresponds to a unique donor, whereas colors indicate a different group, as indicated in the legend.

**Figure 3:**
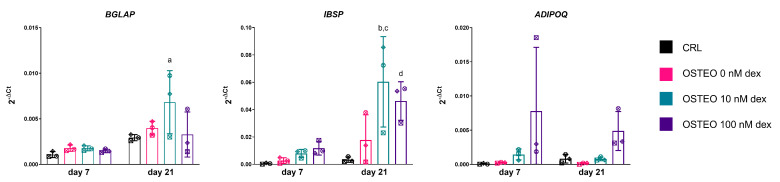
Expression of genes encoding for late markers of differentiation, i.e., osteocalcin (*BGLAP*) and bone sialoprotein (*IBSP*) for osteogenic differentiation, and adiponectin (*ADIPOQ*) as an adipocyte marker. Summary of statistical analysis with a two-way ANOVA with the Tukey post-hoc test. *BGLAP*. a: *p* < 0.05; Osteo 10 nM dex vs. CRL, day 21. *IBSP*. b: *p* < 0.001; Osteo 10 nM dex vs. CRL, day 21. c: *p* < 0.05; Osteo 10 nM dex vs. Osteo 0 nM dex, day 21. d: *p* < 0.05; Osteo 100 nM dex vs. CRL, day 21. *ADIPOQ*: n.s. Each symbol corresponds to a unique donor, whereas colors indicate a different group, as indicated in the legend.

The gene expression levels of key transcription factors (Figure 2) and late markers of differentiation (Figure 3) were analyzed. The results showed that *RUNX2* was not significantly regulated by dex. However, a trend towards a downregulation of *SOX9* with increasing concentrations of dex was observed, which resulted in a significantly higher *RUNX2*/*SOX9* ratio with 100 nM. In line with the identification of adipocyte-like cells within the osteogenic cultures, the levels of *PPARG* were considerably upregulated by dex in a dose-dependent manner, in particular at day 7. Other transcription factors with an involvement in osteogenic differentiation showed no significant change in expression, with a trend toward higher levels of *DLX5* and *SP7* with 10 nM dex at day 7 only, which might reflect a higher activity in early osteogenic commitment (Figure 2).

The expression of late markers of differentiation revealed a peak of *BGLAP* and *IBSP* expression with 10 nM dex at day 21, which was reduced with the higher dose of glucocorticoid. Interestingly, although not significantly, adiponectin appeared to be more expressed in 100 nM dex (Figure 3).

### 2.2. Effect of Dex on RUNX2, SOX9, and PPARG Expression

**Figure 4:**
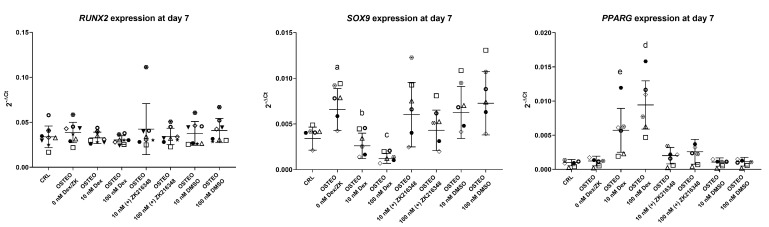
Effect of dex and (+)-ZK 216348 on the expression of *RUNX2*, *SOX9*, and *PPARG* at day 7. N = 8 donors (each represented by a unique symbol). Dimethyl sulfoxide (DMSO) groups were also used as vehicle controls for (+)-ZK 216348. Summary of statistical analysis. *RUNX2*. n.s. *SOX9*. a: *p* < 0.01 OSTEO 0 nM dex/ZK vs CRL, dex groups, 100 nM (+)-ZK 216348. b: *p* < 0.01 OSTEO 10 nM dex vs. 0 nM dex; *p* < 0.05 OSTEO 10 nM dex vs. 100 nM dex, 100 nM (+)-ZK 216348 and DMSO groups. c: *p* < 0.01 OSTEO 100 nM dex vs. CRL, 0 nM and DMSO groups; *p* < 0.05 OSTEO 100 nM dex vs. 10 nM dex and (+)-ZK 216348 groups. ***PPARG***. d: *p* < 0.01; OSTEO 100 nM dex vs. all groups. e: *p* < 0.05; OSTEO 10 nM dex vs. all groups (*p* < 0.01 vs. 100 nM dex).

The early expression of *RUNX2*, *SOX9*, and *PPARG* was studied in a larger number of donors (timepoint: day 7, n = 8). Cultures were also treated for comparison with the same concentrations of (+)-ZK 216348, a synthetic glucocorticoid which shares the transrepressional activity of dex on glucocorticoid receptor but does not stimulate the transactivation pathways [32]. The results confirmed that dex had no effect on the expression of *RUNX2*, while strongly inhibiting *SOX9* and upregulating *PPARG* levels in a dose-dependent manner (Figure 4). The trend for *SOX9* and *PPARG* gene expression was consistent among donors. SOX9 was upregulated in the presence of ascorbic acid and glycerol 2-phosphate alone, and the addition of 10 nM and 100 nM dex strongly suppressed *SOX9* expression. On the contrary, *PPARG* levels were unaltered when ascorbic acid and glycerol 2-phosphate were added, but the presence of dex resulted in a dose-dependent increase in its expression. (+)-ZK 216348 showed no effect on gene expression of either *RUNX2*, *SOX9*, or *PPARG*, although a non-significant trend in *SOX9* downregulation and *PPARG* upregulation can be observed when using higher concentrations of this glucocorticoid receptor agonist.

### 2.3. Two-Stage Osteogenic Differentiation

**Figure 5:**
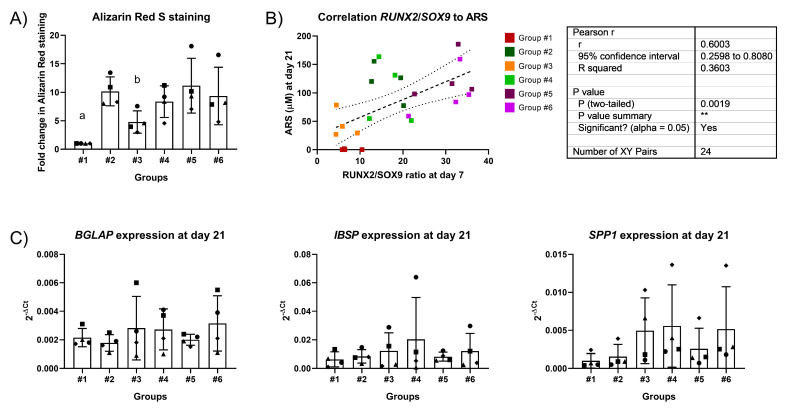
Osteogenic differentiation using different timings and concentration of dex. Each donor is represented by a unique symbol. (**A**) Alizarin Red staining quantification. For each donor, the results are expressed as a fold-change in the intensity of staining compared to the undifferentiated controls. a: *p* < 0.001, #1 vs. #2; *p* < 0.01, #1 vs. #4, *p* < 0.001, #1 vs. #5; *p* < 0.01, #1 vs. #6. b: *p* < 0.05, #3 vs. 2 and #3 vs. 5. (**B**) *RUNX2*/*SOX9* ratio correlation to ARS. Pearson’s correlation was calculated to correlate the *RUNX2*/*SOX9* ratio (2^-ΔCt^ ratio) at day 7 with amount of Alizarin Red staining at day 21, expressed as a fold-change in comparison to the undifferentiated control. The correlation was found to be significant, with a *p*-value of 0.0019. The dashed line represents the line of best fit, of which the equation is reported. The dotted lines represent the 95% confidence interval. (**C**) Gene expression level of late osteogenic markers osteocalcin (BGLAP), integrin-binding sialoprotein (IBSP) and osteopontin (SPP1).

Osteogenic differentiation was further induced using hBMSCs from four additional donors. A two-stage protocol was employed, divided into week 1 and week 2+3. Simply, the protocols differed in the concentration of dex used (Table 1). The Osteo 100 nM group for 21 days was not included at this point. The results showed that reducing the concentrations of dex after the first week of induction did not impair the mineralization potential, as depicted in Figure 5. Indeed, Alizarin Red staining levels remained at comparable levels to when 10 nM dex were maintained for the whole 3 weeks of differentiation. The use of a high dose (100 nM) for the first week did not significantly improve calcium deposition. Interestingly, the correlation between *RUNX2*/*SOX9* ratio at day 7 and Alizarin Red staining at day 21 was maintained even when the differentiation medium was changed after the first week. The expression of late osteogenic markers was reduced in group #5 and improved when dex was completely withdrawn after the first week (group #6), though the results do not reach statistical significance.

### 2.4. Expression Profiles of RUNX2, SOX9, and PPARG

**Figure 6:**
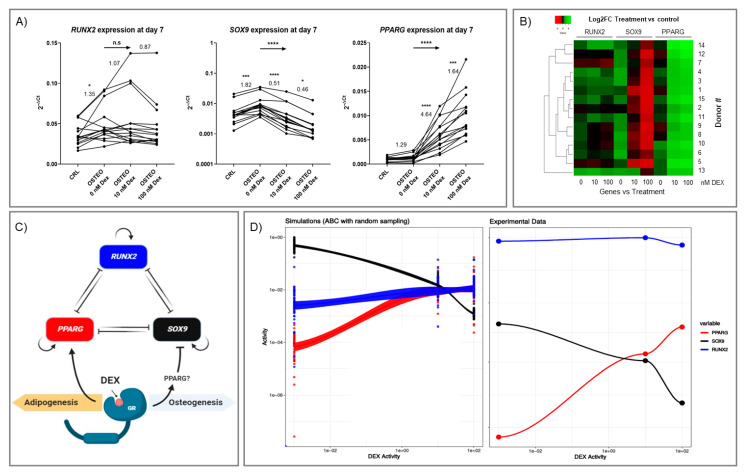
(**A**) Gene expression of *RUNX2*, *SOX9,* and *PPARG* at day 7 during osteogenic differentiation with increasing concentrations of dex. CRL: DMEM-LG, 10% FBS, 1% P/S. OSTEO 0 nM dex: OSTEO medium (DMEM-LG, 10% FBS, 1% P/S, 50 µg/mL ascorbic acid 2-phosphate, 5 mM beta-glycerol phosphate) with no dex added. OSTEO 10 nM dex: OSTEO medium with 10 nM dex. OSTEO 100 nM dex: OSTEO medium with 100 nM dex. n = 15 donors. The before–after graphs depict the expression of *RUNX2*, *SOX9,* and *PPARG* in each individual donor in the different conditions, expressed as 2^-ΔCt^. A repeated measures one-way ANOVA with a post-hoc test for trends confirmed no effect of dex on *RUNX2*, whereas a dose-dependent effect of dex on *SOX9* downregulation (****, *p* < 0.0001) and *PPARG* upregulation (****, *p* < 0.0001) was observed. The numbers above the lines connecting the conditions indicate the average ratio of expression between two different conditions: from left to right, OSTEO 0 nM dex/CRL; OSTEO 10 nM dex/OSTEO 0 nM dex; OSTEO 100 nM dex/OSTEO 10 nM dex. The asterisks indicate if there is a difference between the two conditions compared; * *p* < 0.05; *** *p* < 0.001; **** *p* < 0.0001. The rightward arrows above the graphs summarize the results of the ANOVA test for linear trends in the left-to-right order: n.s., non-significant: **** *p* < 0.0001 (*SOX9* and *PPARG* show a dex dose-dependent change). (**B**) Gene expression profiling of *RUNX2*, *SOX9,* and *PPARG* in response to pro-osteogenic medium with different concentrations of dex (0 nM, 10 nM, 100 nM) in isolated hBMSCs. The data were visualized by a heat-map and the color legend shows the relative expression normalized to the expression level of the respective untreated condition for each gene (Log2FC). The color changes from red (downregulation) to green (upregulation). Clustering analysis indicates the similarities among transcription profiles of donors for the genes of interest. (**C**) Theoretical model of *RUNX2*-*SOX9*-*PPARG* interaction (Figure created with Biorender.com). The basic model was proposed by MacArthur et al. [33]. Based on our experimental results, the model was modified to account for the influence of dex-activated glucocorticoid receptor on gene expression. Dex seemed to exert its effect mainly through *SOX9* and *PPARG* regulation. (**D**) Signaling model compared to experimentally measured activities of *PPARG*, *SOX9*, and *RUNX2*. ABC simulations (left) used to determine the parameters of the theoretical model from random sampling that best represent the experimentally measured activity values (right) of *PPARG*, *SOX9,* and *RUNX2*. Corridor lines represent the R stat_smooth confidence interval over the simulated data points.

Data on *RUNX2*, *SOX9*, and *PPARG* expression were gathered for all the donors involved in this study (Figure 6A). The results from all the donors confirm that dex does not alter *RUNX2* expression, at least at day 7, with upregulation and downregulation of *PPARG* and *SOX9*, respectively.

To assess differences in terms of gene expression levels between samples and similarities among donors’ profiles, the relative transcription levels of the treated samples (osteogenic medium with different concentrations of dex) were normalized to the expression in the respective control group (Log2 of the fold change [Log2FC], see Supplementary Table S2), and the results were represented through hierarchical heatmaps (Figure 6B). Dex at both concentrations upregulated *PPARG* in all donors (Log2FC ≥ 1.47); in the majority of the donors, upregulation was also measured in the osteogenic medium in the absence of dex but at a lower level, except for donors #1, #8, #9, and #12. On the contrary, for *SOX9* a polarization to downregulation was observed in a dose-dependent manner, despite a tendency to be upregulated in the presence of osteogenic medium alone (Log2FC range (0.03–1.47)). Interestingly, donor #13 showed almost no effect at 10 nM dex (Log2FC = 1.18) compared to osteogenic buffer alone (Log2FC = 1.05).

As already observed, *RUNX2* showed the most heterogeneous results among donors. As further emphasized in Figure S3, four main clusters of patients were present, with #5 being most dissimilar to the other donors.

Based on the gene expression results, a simplified theoretical model of the interaction among transcription factors [33] was modified to take the effect of dex into account (Figure 6C). The signaling network was used to perform steady-state simulations (Figure 6D, left) [34,35,36]. The activity profile of the in-silico network was then compared to the experimentally observed activities via approximate Bayesian computation (ABC) [37]. This allows the estimation of the physicochemical parameters underlying the network regulation in the present experimental context.

The simulations successfully captured the trend observed experimentally, indicating a ~50-fold lower degradation rate for *SOX9* as a hallmark of the current observations. However, the proportion of the relative response between *RUNX2*, *SOX9*, and *PPARG* appears to be different. Simulations predict *RUNX2* to have a lower activity than what it is observed experimentally (Figure 6D).

ABC allowed us to estimate model parameter values that led steady-state simulations to match as well as possible with the results observed experimentally (Figure 6A). Figure S1 shows the uniformly random prior distribution of the parameters used as inputs in the ABC procedure. ABC then selected the parameter ranges for which the steady-state model matched the observed experimental values of Figure 6A (see also Figure S2).

The estimated decay rates of *PPARG* and *RUNX2* indicated values in the central to upper range of the sampled space, supporting a relatively faster degradation than *SOX9*, which was restricted to a narrow range at low values (see Figure S2, first row).

The affinities of the inhibitory interaction between *RUNX2*, *SOX9*, and *PPARG* indicated selected values in the central range of the sampled parameters, with exceptions for the affinities of *RUNX2* to *SOX9* and of *SOX9* to *RUNX2*, which were slightly skewed to a higher and a lower parameter range, respectively (Figure S2, second and third rows). This indicated that, in the mutual inhibitory interaction between *RUNX2* and *SOX9*, *SOX9* exerted a stronger effect on *RUNX2* than the opposite reaction (as affinity here was defined as a dissociation binding constant—the smaller the value the stronger the molecular interaction).

Finally, ABC indicated a more important role for the strength of the *SOX9* feedback loop compared to *PPARG* and *RUNX2* feedbacks. *SOX9* feedback more frequently assumed low values (strong feedback) in the simulations selected by ABC to be matching the experimental results (Figure S2, last row).

## 3. Discussion

Despite the physiological regenerative capability of bone and the huge body of research on osteogenic differentiation, the potential for the translation of in vitro results is still quite limited [3] and to date there are no bone-related clinical tissue engineering applications approved that include a cellular component (as per April 2021, see https://www.fda.gov/vaccines-blood-biologics/cellular-gene-therapy-products/approved-cellular-and-gene-therapy-products for FDA approvals, accessed on 19 March 2021) [38]. One of the simplest explanations for this phenomenon is that there are some inherent issues with the experimental protocols that are employed to study in vitro osteogenesis. In this regard, it is striking that the majority of protocols for inducing osteogenic differentiation in bone-marrow-derived mesenchymal stromal cells relies on the use of dex. Even though it has a pro-osteogenic effect, the evidence from the clinical use of glucocorticoids is that it negatively affects bone tissue and vasculature health, with serious sequelae for the patient in the long term. [39] Combining these apparently contradictive effects with the presence of dex in differentiation protocols toward other classical mesenchymal lineages (chondro- and adipogenic), this raised the question of whether dex is the weakest link in the chain.

To answer this question, we started looking at the effect of increasing dex concentrations on pro-osteogenic cultures, focusing on gene expression and final differentiation outcomes. This work provides evidence for an off-target adipocytic differentiation in standard osteogenic cultures of hBMSCs. This effect is induced by dex, which strongly promotes *PPARG* expression in a dose-dependent manner. The presence of pre-adipocyte-like cells, even if it is limited, should warn us that the protocol currently in use needs to be reconsidered because it is not possible to control unwanted cell fate commitment. Even though our standard protocols use 10 nM dex, which showed a more limited presence—but not the absence—of lipid droplets, many papers nevertheless report the use of higher doses of dex for the osteogenic differentiation of hBMSCs. A recent paper also deals with the effect of different concentration of BMSCs in osteogenic and adipogenic differentiation [40]. In that paper, it was shown that the 100-nM dose of dex resulted in a higher level of mineralization in hBMSC osteogenic cultures, suggesting this concentration for the induction of osteogenesis. However, our results so far suggest that a lower concentration of dex (10 nM) might be better to maximize its pro-osteogenic effects, with higher levels of late marker expression (i.e., *BGLAP* and *IBSP*), lower expression of adiponectin, and an overall minor presence of adipocyte-like cells.

The double-edged effect of dex on differentiation has been demonstrated before in murine cells, where it was indeed observed that dex may lead to heterogeneous osteogenic/adipogenic commitment [27]. Notably, it was also suggested that osteoblasts with high *PPARG* expression may switch fate and become adipocytes [28]. In addition, our results are in line with previous reports showing that dex promotes adipose tissue to the detriment of bone formation in bone marrow [10].

Our results also indicate that dex is required to induce downregulation of *SOX9*, and it counteracts the effect of ascorbic acid and glycerol 2-phosphate alone, which conversely upregulated *SOX9* expression. *SOX9* downregulation by dex has been previously reported in cells from the brain system (murine astrocytes [41,42] and neural stem cells [41,43] with 100 nM dex, and in rat hypothalamus [41,44] with a 2 mg/kg dose in vivo) and in the mouse chondroprogenitor cell line ATDC5 (1 µM) [45].

The balance of three key transcription factors for the determination of cell lineage (i.e., *RUNX2* for osteogenic [19], *SOX9* for chondrogenic [46], and *PPARG* for adipogenic differentiation [47]) is pivotal in addressing which fate the cell will undertake. Our group has previously shown that the *RUNX2*/*SOX9* ratio, rather than *RUNX2* alone, is a better predictor of the mineralization potential of hBMSCs due to changes in *SOX9* expression [31]. Here we propose a model which also takes *PPARG* into consideration and shows how its expression is dependent on dex treatment. It is worth highlighting that the effects pointed out by the ABC simulations are in support of a role for *SOX9* that counters the indirect stimulatory effect of dex. According to those simulations, the signal that passes through *SOX9* to *RUNX2,* and consequently stimulates bone formation, relies on the strong *SOX9* feedback and its slow degradation rate, which both act disruptively for the disinhibition pathway of dex ⊣ *SOX9* ⊣ *RUNX2* (where ⊣ represents the inhibition symbol). This is also suggested by the stronger inhibitory interaction of *SOX9* with *RUNX2*, compared to the inverse effect of *RUNX2* ⊣ *SOX9*. On one hand, this ABC model successfully captured the experimental trends of gene expression. On the other hand, the range of the responses were different, which might indicate that the model scheme used to carry out the simulations was oversimplified, and that further signaling components are necessary for a better convergence of the model with the experimental observations.

It is worth mentioning that the present work is relevant for understanding mechanisms underlying intramembranous ossification. Indeed, with this in vitro model, we targeted the direct differentiation of hBMSCs to the osteoblastic lineage. From our results, we cannot infer directly what the effect of dex is during endochondral ossification and how the transcription factors are regulated, as the two processes and the in vitro models are very different. However, exogenous glucocorticoid administration is known to induce long bone growth retardation, probably through several direct and indirect mechanisms that affect chondrocytes and vascular invasion [48]. Dexamethasone is also a common component of chondrogenic differentiation cocktails; intriguingly, we have previously shown that its absence ameliorates the chondrogenic differentiation of synovial derived stem cells induced by TGF-β1 and BMP-2, whereas its presence usually results in higher *PPARG* levels [49].

Further investigation is warranted to better understand the effect of dex on a single-cell level. Indeed, hBMSC cultures comprise a heterogeneous population of cells with different degrees of potential and differentiation. Usually, it is supposed that the relative activity of *RUNX2* versus *PPARG* decides the cell fate commitment toward osteo- or adipogenesis, which are mutually exclusive [50]. Since in our cultures we could observe both types of differentiation at the same time, it is likely that there might be a different response to dex at a single-cell level. Whether dex selects for cells with specific characteristics (e.g., physiologically less osteogenic and with a more adipogenic-committed phenotype) or not is crucial to better understand how dex controls differentiation. Moreover, this may lead us toward understanding how to maintain only the desirable effects of dex (which is, *SOX9* downregulation and osteogenic differentiation), while excluding the activation of off-target pathways leading to adipogenic commitment. This would be fundamental for a proper clinical translation of in vitro osteogenesis results. Another point that requires further studies is identifying which mediators lead to *SOX9* downregulation. Indeed, our results suggest that this effect is mediated mainly by a transactivation pathway, since we could not observe comparable results by treating cells with the same concentrations of (+)-ZK 216348. Therefore, we should aim to understand whether it is *PPARG* that directly induces *SOX9* downregulation alone, or if there is any other actor involved. If the former hypothesis is true, this would mean that the adipogenic and the osteogenic effects of dex are more closely intertwined than expected; therefore, completely new approaches to in vitro osteogenic differentiation should be developed. Finally, our results indicate that there is a high donor-related variation of *RUNX2* expression in response to dex. Clarifying this aspect might be crucial in order to better predict the differentiation potential of hBMSCs. In the present work, age, gender, and cell duplication time in culture did not seem to predict the response of *RUNX2* expression to dexamethasone (data not shown).

In conclusion, here we show that despite its role in inducing osteogenic differentiation via the inhibition of *SOX9* expression, the effects of dex are too broad and an adipogenic program can also be initiated, in at least a subset of cells. We need to rethink how we study differentiation in vitro, with the commonly used standard protocols not necessarily representing the best solution. One alternative might involve the use of two- or multi-stage protocols, with each stage being more adapted to the differentiation stage (induction ≠ mineralization and maturation). In this work, we have tested a two-stage protocol that is still based on the use of dexamethasone, in which we were able to show a slight improvement. A short burst of dexamethasone in the early differentiation commitment is probably more beneficial for the cells, but our hypothesis is that we should avoid dexamethasone completely if we want to improve research in the bone field. Indeed, on one side we show that we can limit the exposure of cells to dex and still achieve good levels of mineralization, and this approach might be further improved by focusing on even earlier time points. On the other hand, this work raises the urgent need to find new strategies and compounds that can induce *SOX9* downregulation, while excluding *PPARG* upregulation.

## 4. Materials and Methods

### 4.1. Cell Isolation and Induction of Osteogenic Differentiation

Isolation of mesenchymal stromal cells from human bone marrow (hBMSCs) was performed as described before [16]. After obtaining full ethical approval (KEK-ZH:2010-0444/0) and with written informed consent, cells from 15 donors were utilized (9 M/6 F; mean age 42 years; age range 15–80; see Figure S4). After isolation, an initial cell density of 3 × 10^3^ cells/cm^2^ was maintained for the hBMSC subculture, and these were grown until passage 2 (p2) in Minimum Essential Medium Eagle—Alpha Modification (α-MEM, Gibco, Thermo Fisher, Zürich, Switzerland) with the addition of 100 μg/mL streptomycin (Gibco), 100 U/mL penicillin (Gibco), 5 ng/mL basic fibroblast growth factor (bFGF, Fitzgerald Industries International, Acton, MA, USA), and 10% MSC-qualified fetal bovine serum (FBS, Pan-Biotech, Aidenbach, Germany). Maintenance of cultures was performed at 37 °C/5% CO_2_, and the medium was refreshed on alternate days.

For osteogenic differentiation, seeding of cells at p3 was performed at a density of 1.5 × 10^4^ cells/cm^2^ on Thermanox coverslips (Nunc, Milian AG, Geneve, Switzerland). The medium was changed after overnight cell attachment; cultures of control (CRL) samples was carried out in Dulbecco’s modified Eagle’s medium (DMEM) with 1 g/L glucose, 100 μg/mL streptomycin, 100 U/mL penicillin, and 10% heat-inactivated FBS (all from Gibco). The addition of 5 mM β-glycerol phosphate, 50 μg/mL ascorbic acid 2-phosphate, and varying concentrations of dexamethasone–cyclodextrin complex (water-soluble formulation) to the control medium was used to induce osteogenic differentiation (all from Sigma-Aldrich, Buchs, Switzerland). Cells were maintained in osteogenic medium with different dex concentrations (0 nM, 10 nM, and 100 nM) for 21 days (n = 3 donors) or with two-stage protocols, as described in Table 1 (n = 4 donors). To maintain stable levels of differentiation factors, the medium was refreshed on alternate days. For RNA isolation, the collection of samples was performed at day 7 and day 21. Furthermore, for evaluating final differentiation outcomes, Alizarin Red S and Oil Red O staining were performed on day 21 to evidence calcium deposition and the presence of lipid droplets, respectively. In a separate experiment (n = 8 donors), the same concentrations of dex, (+)-ZK 216348 (Axon Medchem BV, Groningen, The Netherlands), or dimethyl sulfoxide (DMSO) as a vehicle control were tested in osteogenic differentiation, and RNA was isolated after 7 days for gene expression analysis.

### 4.2. Alizarin Red Staining and Quantification

Samples at day 21 were fixed in 4% neutral buffered formalin and stained with a 40 mM, pH 4.2 solution of Alizarin Red S (Sigma-Aldrich). After extensive washing to remove unbound staining, mosaic pictures were created with an Evos2 microscope (ThermoFisher). Subsequently, Alizarin Red was eluted from cultures, using the cetylpyridinium chloride method, as previously described [51], and quantification was performed by measuring absorbance at 540 nm. 

### 4.3. Oil Red O Staining and Quantification

Samples at day 21 were fixed in 4% neutral buffered formalin, washed with 2-propanol, and stained with an Oil Red O working solution (0.5% Oil Red O in 2-propanol, 60% in H_2_O). After a further wash in 2-propanol, the cultures were washed and maintained in H_2_O for imaging. Pictures were acquired using an Axiovert 40 inverted microscope, CP-Achromat 5×/0.12 objective, equipped with an Axiocam 105 color camera (Carl Zeiss Microscopy GmbH, Jena, Germany). At least 10 representative pictures were taken for further image analysis. Images were analyzed with Fiji/ImageJ software (NIH, Bethesda, MD, USA). Briefly, raw images were imported and converted to RGB. A color threshold was applied to select stained cells, then the area covered by the selection was measured (in µm^2^). The percentage out of the total area was calculated. Final results were calculated as the average % area of the 10 images.

### 4.4. Isolation of RNA

Standard TRIreagent extraction (Molecular Research Center Inc., Cincinnati, OH, USA) with 1-bromo-3-chloropropane (Sigma-Aldrich) was used for the isolation of total RNA. After phase separation, the precipitation of RNA was carried out from the aqueous phase by adding 2-propanol (Sigma-Aldrich). The RNA pellet was washed with 75% EtOH and finally reconstituted with diethylpyrocarbonate (DEPC)-treated H_2_O. A NanoDrop 1000 (Thermo Fisher) was used to measure the total RNA concentration and purity was evaluated via assessment of the A260/280 and A260/230 ratios.

### 4.5. Gene Expression Analysis

For total gene expression, TaqMan Reverse Transcription reagents (Applied Biosystems, Foster City, CA, USA) were used to synthesize cDNA from 1000 ng of total RNA, as per the recommendations of the manufacturer. Differentiation markers in osteogenic differentiation (see Table S1) were analyzed using qPCR. TaqMan Gene Expression Master Mix (Applied Biosystems) in a QuantStudio 7 Flex Real-Time PCR system (Applied Biosystems) was used to achieve the amplification of target genes by applying the following protocol: 2 min at 50 °C; 10 min at 95 °C; 40 cycles of 15 sec at 95 °C, 1 min at 60 °C. TaqMan gene expression assay (Thermo Fisher) or custom-designed primers and probes (Microsynth AG, Balgach, Switzerland) were used to test the genes of interest (Table S1). Results were expressed as 2^-ΔCt^, with *RPLP0* used as a reference gene.

### 4.6. Approximate Bayesian Computation Simulations

A network interaction model, including four molecular species, one activation, seven inhibitory, and three positive feedback reactions, was used as an input to carry out steady-state simulations [36,52]. The method used basal/decay rates and Hill-type transfer functions for species and reactions, respectively, and solved ordinary differential equations that determined the dynamics of the system at the steady state, to be compared to the results obtained experimentally [37]. Interactions between species were assumed to be linear by making the Hill coefficient of each reaction equal to one, as for the other model parameters, unless otherwise specified.

Independently, the basal activities of *RUNX2*, *SOX9*, and *PPARG* were kept as fixed parameters in the simulations, at the values measured experimentally corresponding to 0 nM dex stimulation. Dex activity was used as a corresponding measure of the experimental concentrations, by means of scaling experimental and simulated data (both were scaled to 1 by dividing by their respective maximal value). All other parameters that determine the decay of *RUNX2*, *SOX9*, and *PPARG*, as well as the affinities of their interactions, including the strength of the positive feedback loops, were varied according to a random scheme. The range of decay rates was [0.001–1], whereas the range of affinities was [0.001–100]. The steady-state values generated by the model according to this sampling scheme constituted the prior probability distribution used for ABC (Figure S1).

Full data were used instead of summary statistics to carry out ABC using the Euler distance method between the experimental data per each dex concentration, and the steady state of the simulated data. The CRAN ABC package [53] was used, with a tolerance of 0.005, using the rejection method to infer the posterior probability distribution (Figure S2) and consequently to estimate model parameters that best represented the experimental results.

### 4.7. Heatmap Generation and Statistical Analysis 

Differential expression levels of genes were calculated as Log2 (ratios) of hBMSCs treated with pro-osteogenic medium +/− dex at different concentrations (10 nM, 100 nM), normalized to their respective untreated condition (control = CTRL). Genes with a Log2FC ≥ 0.585 (corresponding to a 1.5-fold change) were considered to be differentially expressed. Hierarchical clustering on donors and colorimetric representation of the relative transcriptional profiles were represented through heat-maps generated by Heatmapper [54] (https://github.com/WishartLab/heatmapper, accessed on 19 March 2021), setting the Pearson correlation as the distance measure and average linkage as the clustering method.

Statistical analysis was performed using GraphPad Prism v.8 (GraphPad Software, San Diego, CA, USA) for the analysis of gene expression with increasing doses of dex (or (+)-ZK 216348). After testing for a Gaussian normal distribution with the Shapiro–Wilk normality test, a repeated-measure one- or two- way Analysis of Variance (ANOVA) with the Geisser–Greenhouse correction was applied, followed by Tukey’s multiple comparison test to compare the means between the different groups or by testing for linear trends. Matching was effective for the genes tested, with a *p* = 0.0002 for *RUNX2*, *p* < 0.0001 for *SOX9,* and *p* = 0.0020 for *PPARG*. Pearson’s coefficient was calculated to test possible correlations.

## Figures and Tables

**Table 1:** Scheme of the two-stage protocols for osteogenic differentiation. The osteogenic differentiation medium (OSTEO) contains 5 mM β-glycerol phosphate and 50 μg/mL ascorbic acid 2-phosphate, with the indicated amount of dex. CRL medium refers to the growth medium containing DMEM, 10% FBS, and penicillin/streptomycin only.

Group	Week 1	Week 2 + 3
#1	CRL medium	CRL medium
#2	OSTEO 10 nM dex	OSTEO 10 nM dex
#3	OSTEO 0 nM dex	OSTEO 0 nM dex
#4	OSTEO 10 nM dex	OSTEO 0 nM dex
#5	OSTEO 100 nM dex	OSTEO 10 nM dex
#6	OSTEO 100 nM dex	OSTEO 0 nM dex

## Data Availability

All data and materials used in the analysis are available to any researcher for purposes of reproducing or extending the analysis.

## Supplementary Materials

The following are available online at https://www.mdpi.com/article/10.3390/ijms22094785/s1, Figure S1: Prior (uniformly random) distributions. Figure S2: Posterior (selected by ABC) distributions. Figure S3: Gene expression profiling and donor clustering analysis of *RUNX2* in response to pro-osteogenic medium with different concentrations of dex (0 nM, 10 nM, 100 nM) in isolated hBMSCs. Figure S4: Summary of BMSC donors used in each experiment. Table S1: List and details of primers and probes used for gene expression analysis. Table S2: complete dataset of Log2FC values of *RUNX2*, *SOX9*, and *PPARG* expression.
