# Supplementary material for: Dexamethasone Induces Changes in Osteogenic Differentiation of Human Mesenchymal Stromal Cells via SOX9 and PPARG, but Not RUNX2
Source: Int J Mol Sci. 2021 Apr 30;22(9):4785. doi: 10.3390/ijms22094785 (PMC8124248; doi:10.3390/ijms22094785)
Supplement: Supplementary file 1 [file ijms-22-04785-s001.zip › ijms-1170405-supplementary.pdf]

Supplementary material to:

Elena Della Bella, Antoine Buetti-Dinh, Ginevra Licandro, Paras Ahmad, Valentina Basoli, Mauro Alini, Martin James Stoddart. Dexamethasone induces changes in osteogenic differentiation of human mesenchymal stromal cells via SOX9 and PPARG, but not RUNX2.

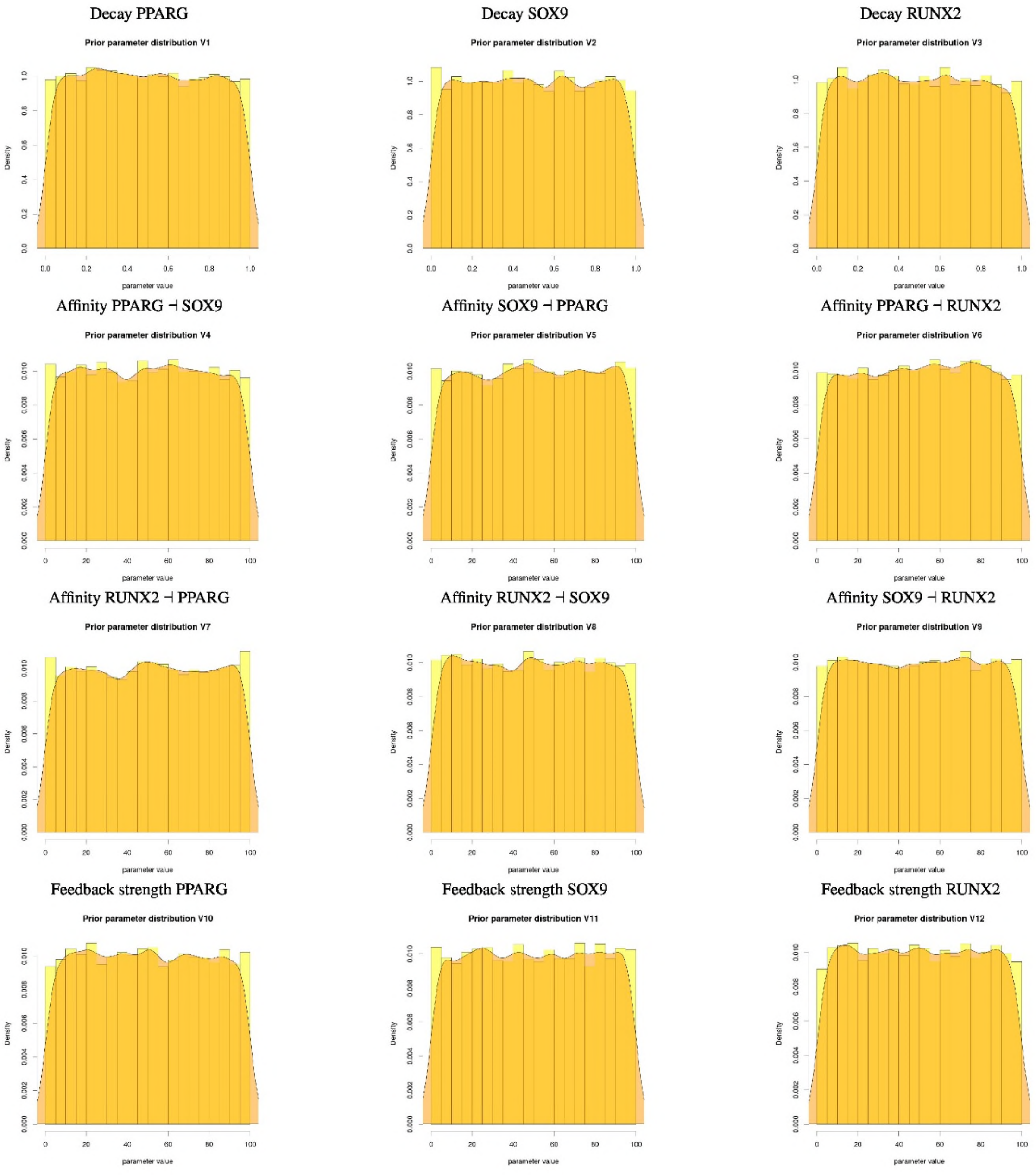

Figure S1. Prior (uniformly random) distributions. The number of bins is estimated with the R Sturges method and the density estimation shown in continuous line. Decay indicates a first order reaction rate of species degradation, while affinity and feedback strength are defined as dissociation binding constants, the smaller the value the stronger the molecular interaction.

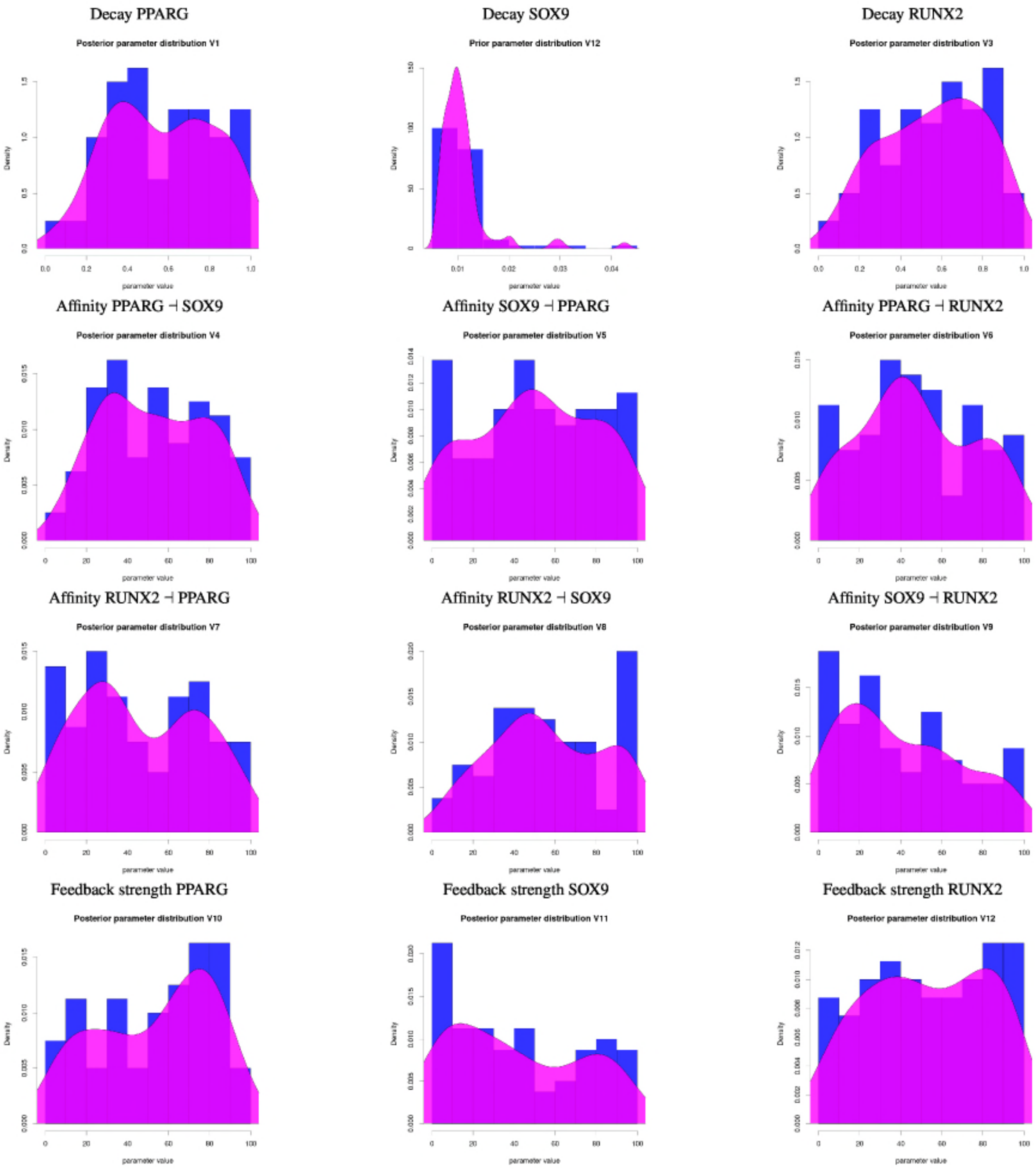

Figure S2. Posterior (selected by ABC) distributions. The number of bins is estimated with the R Sturges method and the density estimation shown in continuous line. Decay indicates a first order reaction rate of species degradation, while affinity and feedback strength are defined as dissociation binding constants, the smaller the value the stronger the molecular interaction.

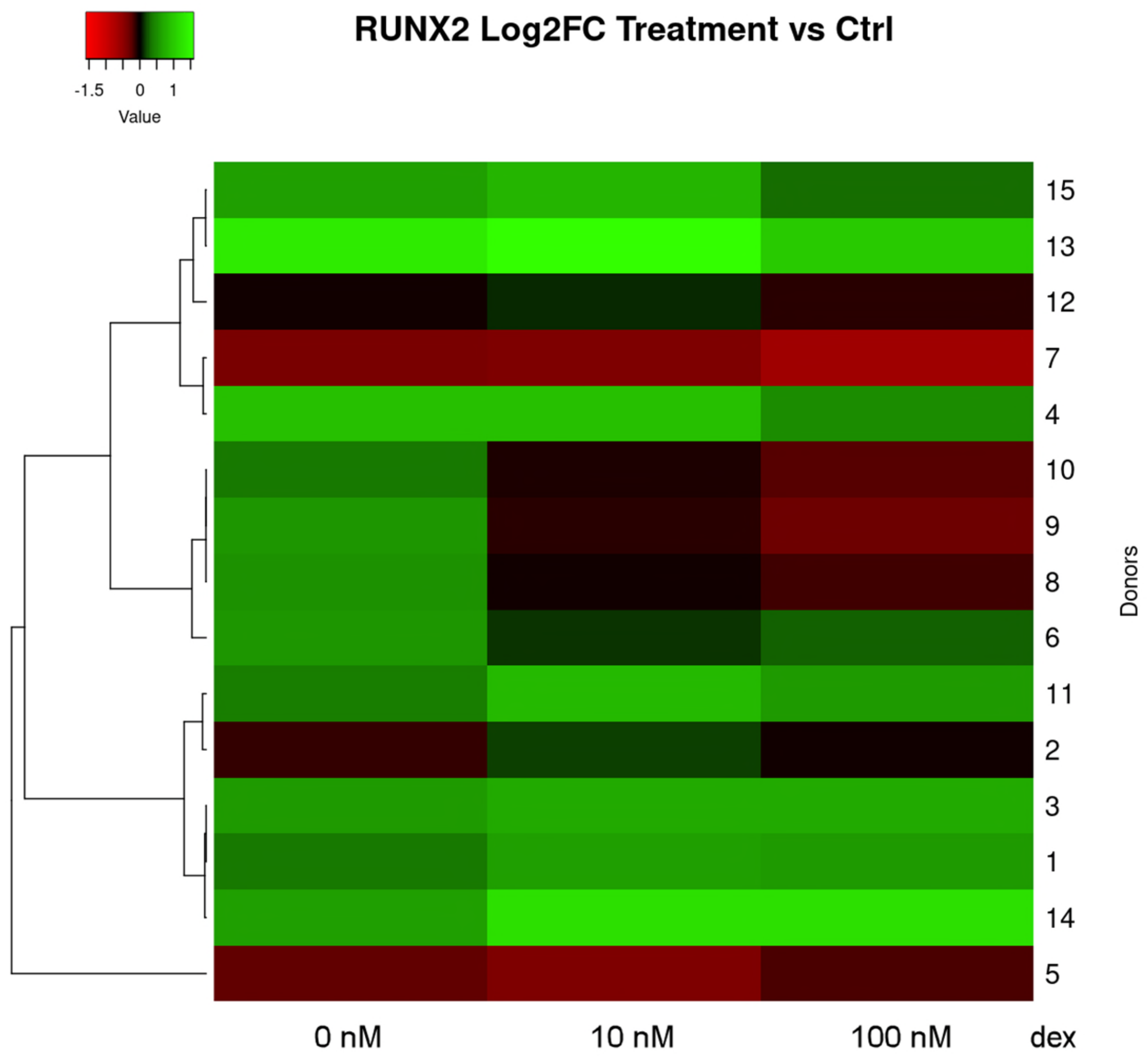

Figure S3. Gene expression profiling and donor clustering analysis of RUNX2 in response to pro-osteogenic medium with different concentration of dex (0 nM, 10 nM, 100 nM) in isolated hBMSCs. The data were visualised by a heat-map and colour legend shows the relative expression normalised to the expression level of the respective untreated condition (Log2FC).

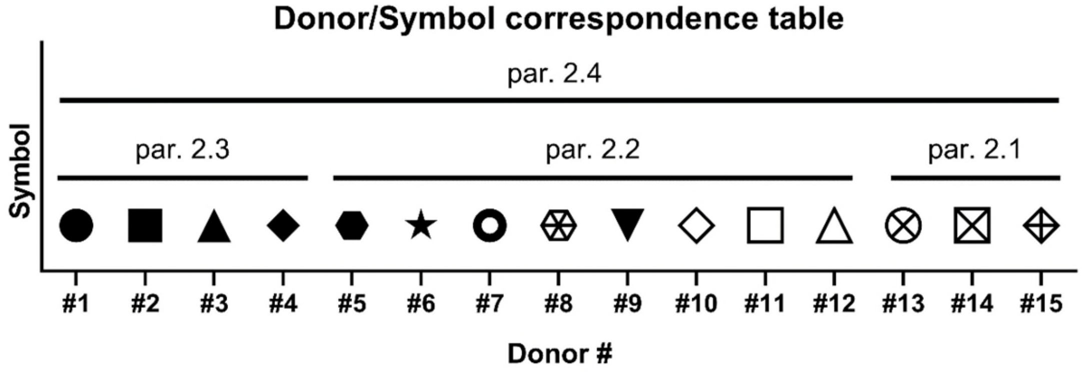

Figure S4. Summary of BMSC donors used in each experiment, with reference to the paragraph where the results are showed. Each donor is represented throughout the manuscript using a unique symbol.

Table S1: List and details of primers and probes used for gene expression analysis

GENE	ASSAY DETAILS
BGLAP*	Forward primer: 5'-AAGAGACCCAGGCGCTACCT-3'<br>Reverse primer: 5'-AACTCGTCACAGTCCGGATTG-3'<br>Probe: 5'-ATGGCTGGGAGCCCCAGTCCC-3'
DLX5*	TaqMan Gene Expression Assay Hs00193291_m1
IBSP*	TaqMan Gene Expression Assay Hs00173720_m1
PPARG**	TaqMan Gene Expression Assay Hs00234592_m1
RPLP0*	Forward primer: 5'-TGGGCAAGAACACCATGATG-3'<br>Reverse primer: 5'-CGGATATGAGGCAGCAGTTTC-3'<br>Probe: 5'-AGGGCACCTGGAAAACAACCCAGC-3'
RUNX2*	Forward primer: 5'-AGCAAGGTTCAACGATCTGAGAT-3'<br>Reverse primer: 5'-TTTGTGAAGACGGTTATGGTCAA-3'<br>Probe: 5'-TGAAACTCTTGCCTCGTCCACTCCG-3'
RUNX3**	TaqMan Gene Expression Assay Hs01091094_m1
SOX9**	TaqMan Gene Expression Assay Hs00165814_m1
SP7*	Forward primer: 5'-CCTGCTTGAGGAGGAAGTTCA-3'<br>Reverse primer: 5'-GGCTAGAGCCACCAAATTTGC-3'<br>Probe: 5'-TCCCCTGGCCATGCTGACGG-3'

** Custom sequences (synthesized from MicroSynth). Reporter dye: 6-carboxyfluorescein (FAM); quencher: 6-carboxy-N, N, N´, N´-tetramethylrhodamine (TAMRA). ** Commercially available assays (Thermo Fisher) Reporter dye: FAM; quencher: non-fluorescent quencher, minor groove binder (NFQ-MGB).*

Table S2

# Log2FC values (treatment vs CRL)

Donor	*RUNX2* _0nM	*RUNX2* _10nM	*RUNX2* _100nM	*SOX9* _0nM	*SOX9* _10nM	*SOX9* _100nM	*PPARG* _0nM	*PPARG* _10nM	*PPARG* _100nM
**#1**	0.364529598	0.621267764	0.586432389	0.497499659	-1.087462841	-1.86507042	-0.277984747	2.394440595	2.911839812
**#2**	-0.129691077	0.18651203	-0.03970971	0.033947332	-0.637429921	-1.691877705	0.309104055	1.501749132	2.43248647
**#3**	0.59400764	0.72164092	0.70169551	1.118181426	-0.357552005	-1.657112286	0.355094959	2.287980763	2.77830639
**#4**	0.893084796	0.915864665	0.484411202	1.296981738	-0.175086707	-1.428843299	0.602036014	2.186998515	2.836501268
**#5**	-0.259298465	-0.406886268	-0.204629595	0.540120826	-1.297084024	-1.968135501	0.563900885	3.698025165	4.103059696
**#6**	0.545497746	0.15560533	0.256818974	1.471675214	-0.327574658	-0.752072487	0.771871209	2.379643593	2.837874609
**#7**	-0.358159905	-0.401964873	-0.612654235	0.943842821	0.164386818	-0.97184896	0.012490944	2.299168192	3.339385292
**#8**	0.519275331	-0.038033955	-0.16211124	1.143153773	-0.720909999	-1.616440732	-0.143835773	2.189680297	2.492554967
**#9**	0.541295852	-0.109828759	-0.297993878	0.928446739	-0.462343214	-1.804449191	-0.38466385	1.475158492	2.480668473
**#10**	0.375318145	-0.090521714	-0.242318251	1.013610432	-0.602158116	-1.612574732	0.950531324	2.781654412	3.619663592
**#11**	0.380828674	0.829698126	0.600667401	0.947313575	-0.129837438	-1.301380717	0.387023123	2.299560282	3.584962501
**#12**	-0.055210187	0.107324073	-0.103126114	0.919780107	-0.274174963	-1.746243408	-0.378511623	3.132450296	4.587272661
**#13**	1.339103239	1.583521966	1.006464664	1.178337241	1.049753035	-0.341036918	0.610588308	2.433820975	2.661739794
**#14**	0.620506618	1.222392421	1.228687828	0.746643508	0.259562214	-0.689212463	0.289506617	4.505326385	5.584294432
**#15**	0.626907035	0.792283666	0.3082246	0.828939338	-0.511610759	-1.906242048	0.648014671	3.007417472	3.879867422

Log2FC(1.5)
0.584962501

#Differentially espressed genes (DEG) with Log2FC >0.5849
#or <-0.5849 are highlighted in green
#corresponding to a FC threshold of 1.5

# $2^{-\Delta Ct}$ values (normalized to *RPLP0*)

Donor	*RUNX2* _CRL	*RUNX2* _0nM	*RUNX2* _10nM	*RUNX2* _100nM	*SOX9* _CRL	*SOX9* _0nM	*SOX9* _10nM	*SOX9* _100nM	*PPARG* _CRL	*PPARG* _0nM	*PPARG* _10nM	*PPARG* _100nM
**#1**	0.0327	0.0421	0.0503	0.0491	0.0051	0.0072	0.0024	0.0014	0.00097	0.0008	0.0051	0.0073
**#2**	0.0442	0.0404	0.0503	0.043	0.0042	0.0043	0.0027	0.0013	0.00113	0.0014	0.0032	0.0061
**#3**	0.0265	0.04	0.0437	0.0431	0.0041	0.0089	0.0032	0.0013	0.00086	0.0011	0.0042	0.0059
**#4**	0.0203	0.0377	0.0383	0.0284	0.0035	0.0086	0.0031	0.0013	0.00112	0.0017	0.0051	0.008
**#5**	0.03471	0.029	0.02618	0.03012	0.00403	0.00586	0.00164	0.00103	0.00092	0.00136	0.01194	0.01581
**#6**	0.0252	0.03678	0.02807	0.03011	0.00128	0.00355	0.00102	0.00076	0.00147	0.00251	0.00765	0.01051
**#7**	0.05786	0.04514	0.04379	0.03784	0.00406	0.00781	0.00455	0.00207	0.00115	0.00116	0.00566	0.01164
**#8**	0.04074	0.05839	0.03968	0.03641	0.00417	0.00921	0.00253	0.00136	0.00137	0.00124	0.00625	0.00771
**#9**	0.02974	0.04328	0.02756	0.02419	0.00248	0.00472	0.0018	0.00071	0.00141	0.00108	0.00392	0.00787
**#10**	0.03305	0.04287	0.03104	0.02794	0.00211	0.00426	0.00139	0.00069	0.00089	0.00172	0.00612	0.01094
**#11**	0.01675	0.02181	0.02977	0.0254	0.00488	0.00941	0.00446	0.00198	0.00039	0.00051	0.00192	0.00468
**#12**	0.03276	0.03153	0.03529	0.0305	0.00416	0.00787	0.00344	0.00124	0.00026	0.0002	0.00228	0.00625
**#13**	0.0334	0.0845	0.1001	0.0671	0.0057	0.0129	0.0118	0.0045	0.00186	0.00284	0.01005	0.01177
**#14**	0.0588	0.0904	0.1372	0.1378	0.0208	0.0349	0.0249	0.0129	0.00045	0.00055	0.01022	0.02159
**#15**	0.05966	0.09213	0.10332	0.07387	0.01668	0.02963	0.0117	0.00445	0.00097	0.00152	0.0078	0.01428
